# Recent advances in understanding *Candida albicans *hyphal growth

**DOI:** 10.12688/f1000research.18546.1

**Published:** 2019-05-21

**Authors:** Robert A. Arkowitz, Martine Bassilana

**Affiliations:** 1 Université Côte d'Azur, CNRS, Inserm, Institute of Biology Valrose, Parc Valrose, Nice, France

**Keywords:** morphogenesis, signaling pathways, membrane traffic, secretion, Spitzenkörper, host-interactions, cellular organization

## Abstract

Morphological changes are critical for the virulence of a range of plant and human fungal pathogens. *Candida albicans* is a major human fungal pathogen whose ability to switch between different morphological states is associated with its adaptability and pathogenicity. In particular, *C. albicans* can switch from an oval yeast form to a filamentous hyphal form, which is characteristic of filamentous fungi. What mechanisms underlie hyphal growth and how are they affected by environmental stimuli from the host or resident microbiota? These questions are the focus of intensive research, as understanding *C. albicans* hyphal growth has broad implications for cell biological and medical research.

## Introduction

**Figure 1.**
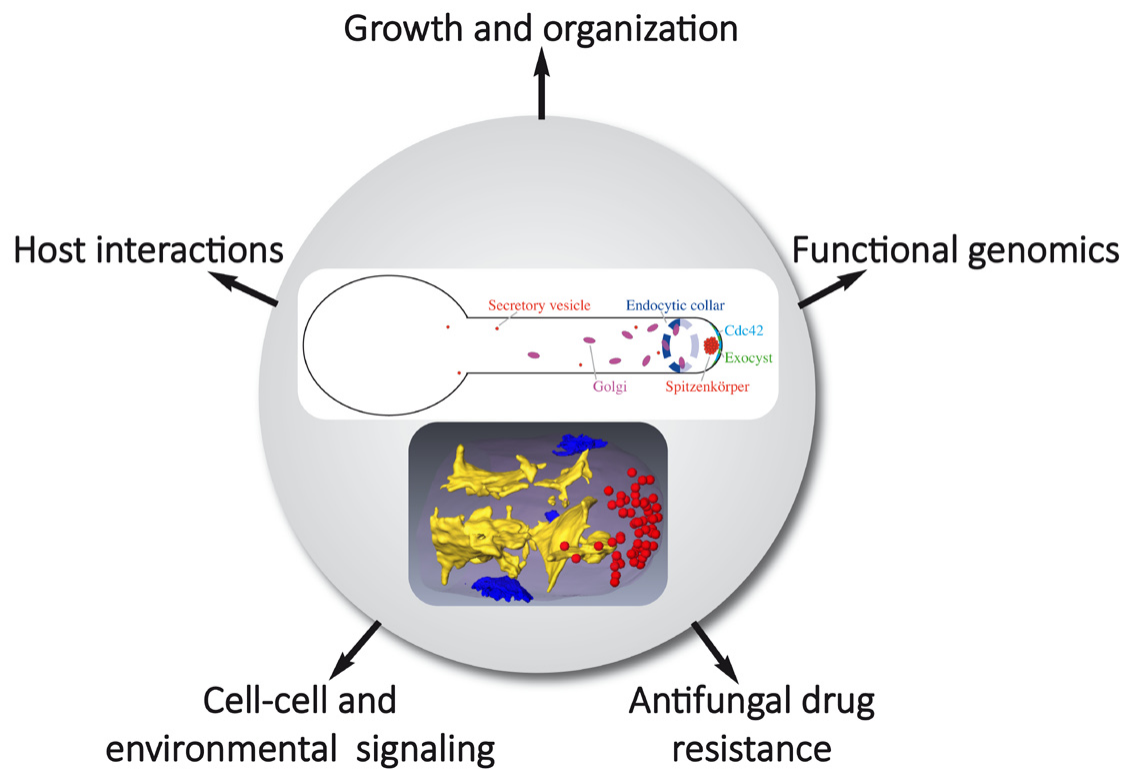
Schematic highlighting *Candida albicans* hyphal organization and studies of morphological transition in different processes and at different levels. The upper panel shows membrane compartments of the exocytic and endocytic pathways focusing on compartments discussed in the review. Endoplasmic reticulum and endosomes, for example, are not shown. The lower panel, reproduced from Weiner *et al*. ^33^, illustrates a segmented three-dimensional dataset from focused ion beam/scanning electron microscopy tomography of a hyphal tip with internal membranes (yellow), secretory vesicles (red), and sites of endocytosis (blue).

Morphology changes occur in a range of human fungal pathogens upon interaction with the host ^1^. In response to different host signals, *Candida albicans* switches from the yeast form to a hyphal form, a cell shape characteristic of filamentous fungi, such as *Aspergillus nidulans* and *Neurospora crassa* ^2– 4^. However, hyphal cells of *C. albicans* are different from those of these organisms with respect to shape/diameter and extension rate (10- to 100-fold slower with this fungal pathogen). Furthermore, in these filamentous fungi, microtubules are critical for hyphal growth, a striking difference with *C. albicans*, in which microtubules do not play a prominent role ^5^. *C. albicans* is an opportunistic human fungal pathogen and a number of studies have linked the switch from yeast to hyphal form with pathogenicity, whether during superficial or systemic infections ^6– 10^. This brief review presents an update of research from the past 2 to 3 years on *C. albicans* technological advances, cell signaling, host interactions, and membrane traffic and puts an emphasis on hyphal growth ( Figure 1).

## Technological advances

In the past several years, technological advances have opened a range of new possibilities in *C. albicans* research. Specifically, the majority of approaches have opened our horizons with respect to large-scale analyses of fungal pathogen function, including a major thrust coming from clustered regularly interspaced short palindromic repeat (CRISPR)-based tools that have particularly revolutionized genome manipulation in genetically less accessible fungi, such as the diploid *C. albicans* ^11, 12^. Other notable approaches that are changing how we work with and view this fungal pathogen include experimental or micro-evolution approaches ^13– 15^, in particular with respect to host niche environments. In addition, large-scale approaches, such as population and genetic diversity analyses via genome sequences of large numbers of isolates ^16^, and the establishment of genomic platforms that facilitate the study of gene function at a genome-wide level ^17– 20^ pave the way for future multi-omic studies.

The application of CRISPR-based methods to *C. albicans* in 2015 was a major step in facilitating molecular genetics in this less genetically tractable fungus ^21^ and opened a myriad of possibilities for studying gene function, including marker recycling ^22, 23^, a “gene drive array” platform for genetic interaction ^12^, rapid gene concatenation for genetic rescue of multi-gene mutants ^24^, and gene regulation ^25, 26^. Overall, *C. albicans* CRISPR-based methods have been substantially optimized ^11, 12, 23, 25– 28^ and now facilitate a range of gene functional analyses up to a genome-wide scale.

Experimental or micro-evolution approaches are particularly powerful tools when applied to opportunistic pathogens ^29^. These approaches have been used initially to identify mutations that restore filamentation in a non-filamentous mutant within macrophages ^30^ and more recently to investigate drug resistance ^14^, host niche-specific mutations ^13^, and the emergence of mutualism between host and fungus ^15^. These approaches, coupled with whole-genome sequencing and other genome-wide methods, are extremely useful when applied to a diploid commensal that can undergo a panoply of genome rearrangements with far-reaching consequences.

The application of novel large-scale approaches, as well as the refinement and optimization of existing methods to gene function analyses in *C. albicans*, will undoubtedly promote a deeper understanding of this fungal pathogen. Chemical inhibitors and chemogenomic profiling have been used to identify genes involved in enhanced antifungal drug sensitivity or resistance ^31^ and novel inhibitors of morphogenesis ^32^.

Genome sequencing and comparative genomics of 182 worldwide *C. albicans* isolates have revealed evidence of gene flow and a highly clonal lineage that has undergone substantial pseudogenization ^16^. In 2018, three major studies highlighted advances made possible by new gene function platforms and tools ^17– 19^. Two groups took advantage of powerful transposon-based approaches, coupled with a stable haploid *C. albicans* derivative, to probe essential genes, generate a comprehensive set of mutants in this fungus, and carry out genome-wide screens ^17, 19^. These studies yielded important information on gene essentiality and azole resistance in this fungal pathogen. Extensive effort was also invested in generating a genomic platform centered on an ORFeome collection representing the majority of open reading frames (ORFs) in Gateway donor vectors, together with a wide range of expression vectors ^18^ facilitating genome-wide overexpression analyses and protein–protein interaction studies ^16, 20, 34^. Together, these new technologies have facilitated recent advances in hyphal growth signaling, host interactions, and membrane traffic.

## Hyphal growth signaling

In the past 2 to 3 years, a range of studies have investigated hyphal growth signaling in *C. albicans* ^35^. These studies have made significant advances, in particular in the areas of amino acid inducers of the hyphal transition ^36, 37^, gaseous sensing and signaling ^38– 42^, and reactive oxygen and oxidative stress signaling ^43– 45^. Extensive analyses of filamentation programs revealed media-independent genetic requirements for filamentation, in particular *RIM101* (pH-dependent pathway) and *GPA2* (Gα functioning in the cAMP/PKA pathway), in addition to a core transcriptional profile ^46^. Also, an investigation into the cAMP requirement for hyphal morphogenesis showed that basal levels of cAMP are sufficient for hyphal formation in response to *N*-acetylglucosamine (GlcNAc), suggesting that cAMP-independent signals are also important for hyphal induction ^47^. Both G _1_ and S phase arrest can induce filamentous growth and this has been shown to require the cAMP/PKA pathway ^48^.

Nutrient deprivation triggers hyphal development in *C. albicans*, and various amino acids have been shown to be critical for this transition. The groups of Van Dijck ^36^ and Ljungdahl ^37^ investigated cAMP/PKA-dependent morphogenesis that is triggered by arginine, ornithine, proline, and methionine metabolism. For these different amino acids, induced expression of amino acid permease genes is critical, with the former three amino acids being metabolized in the mitochondria, resulting in elevated ATP levels that appear to increase activation of the Ras1/cAMP/PKA pathway. With respect to methionine, it is converted to *S*-adenosyl methionine (SAM) that is subsequently decarboxylated and the resulting amino-propyl group is converted to polyamines that have been shown to activate adenylate cyclase.

Although a number of studies have previously analyzed the roles of oxygen and CO _2_ signaling in hyphal development, there has been little attention to nitric oxide (NO) signaling in this process. Koch *et al.* examined a metabolic checkpoint for the yeast-to-hypha transition that is regulated by endogenous NO signaling and their results indicate that sufficient endogenous NO releases Nrg1 repression of this transition ^38^. Three recent studies have shed light on how the tricarboxylic acid (TCA) cycle regulates CO _2_ signaling ^42^, how a phosphatase–kinase pair controls CO _2_-responsive Ume6 phosphorylation and stability ^39^, and have identified a link between CO _2_ sensing and lipid/Pkh1/2 kinase signaling during hyphal development ^41^. In the first of these studies, the authors used a library of TCA metabolic pathway mutants to show that the TCA cycle plays a critical role in regulating CO _2_ sensing and hyphal development ^42^. Lu *et al*. carried out a genetic screen to determine the CO _2_ signaling pathway that regulated Ume6 stability and found that a kinase–phosphatase couple controlled the CO _2_ response of this transcription factor that is crucial for hyphal elongation ^39^. A screen in *Saccharomyces cerevisiae* for mutants that regulate the transcription factor Cst6 ( *C. albicans* homolog Rca1), which activates the carbonic anhydrase *NCE103* in a CO _2_-dependent fashion, identified the kinase Sch9 ^41^. The authors went on to show that Sch9 phosphorylates the transcription factor Rca1 in *C. albicans* and that it links CO _2_ adaptation to lipid signaling via Pkh1/2.

The production of reactive oxygen species (ROS) during *C. albicans* morphogenesis plays an important role in pathogenicity. The conserved heat shock factor-like transcriptional regulator Skn7 is critical for filamentous growth and protection from the accumulation of intracellular ROS in these conditions ^43^. Interestingly, a member of the NADPH oxidase (NOX) family, Fre8, was recently shown to produce a ROS burst during morphogenesis, which is particularly important in the animal host ^45^. Recent studies by Liu *et al.* have shown that inhibition of the major high-affinity phosphate importer, Pho84, sensitized *C. albicans* to oxidative stress via inducing ROS accumulation through activation of TOR (target of rapamycin) signaling ^44^. In addition to these environmental conditions, quorum-sensing molecules, such as farnesol, regulate the morphological transition, and recent work proposed that the response of *C. albicans* to farnesol is influenced by Eed1, a protein critical for hyphal growth maintenance ^49^. Together, these different advances in hyphal growth signaling highlight the important role of hyphal development in host niches and in response to a range of relevant host signals.

## Host interactions

The microbiota is thought to, in part, restrict the fungus to the commensal state ^50^. Of note, the GUT (gastrointestinally induced transition) cells, which are postulated to be a specialized commensal form in the mammalian gastrointestinal (GI) tract, are less virulent in a mouse bloodstream infection model ^51, 52^. The alteration of the balance between commensalism and pathogenicity in the presence of the gut microbiota is associated with mutations in *C. albicans* transcription factors required for white-opaque switching and filamentation, such as Efg1, Wor1, and Flo8 ^15, 52, 53^. Furthermore, *C. albicans* strains that are hyperfit in the antibiotic-treated or germ-free mouse gut tend to be deficient in hyphal morphogenesis ^53, 54^, yet the observation that a hyperfit *ume6* mutant has a ratio of yeast and hyphae similar to that of the wild-type strain in the mouse GI tract would argue that cell shape per se does not determine commensal fitness ^55^. Using an experimental system based on long-term GI tract colonization of mice, a recent work nicely demonstrated that in the absence of microbiota *C. albicans* evolves into strains that lose their ability to form hyphae ^15^. Interestingly, this study additionally shows that priming naïve mice with the gut-evolved strains resulted in a broad cross-protection against *Aspergillus fumigatus*, *Staphylococcus aureus*, or *Pseudomonas aeruginosa*.

Host defense also includes the epithelial physical barrier and host immune cells, such as macrophages. Hyphal growth is associated with mechanical forces during the interaction of *C. albicans* with such host cells ^56^. The relative contribution of these mechanical forces to host cell damage, compared with other hyphal attributes, is an area of active investigation. Mechanical forces appear to be sufficient to penetrate epithelial cells even in the absence of secreted factors, such as the toxin candidalysin, encoded by *ECE1* and therefore secreted only by hyphae ^57^. Indeed, *ece1*Δ/Δ mutant hyphae can invade intestinal epithelial cells without causing damage, yet optimal damage induction requires a combination of hypha formation and candidalysin secretion ^58^. The ability to undergo yeast-to-hypha morphogenesis and the cell wall composition are also important determinants in the macrophage– *C. albicans* interaction ^59^. Recently, *C. albicans* escape from the phagolysosome was proposed to rely directly on physical rupture ^60^. *C. albicans* cells induce macrophage cell death via pyroptosis, a caspase-1–dependent programmed cell death ^61, 62^, and it was proposed that activation of the inflammasome is a consequence of this phagolysosome rupture via the yeast-to-hypha transition ^60^. However, this proposal was challenged by another study, which showed that rupture is not a prerequisite for inflammasome activation, as a collection of genes enabled activation of macrophage pyroptosis independently of effects on morphogenesis, and cell wall remodeling was a major determinant ^63^. The role of candidalysin in the phagocyte inflammatory and damage response to *C*. *albicans* hyphae was recently investigated and this toxin appears to trigger inflammasome activation ^64^. Thus, how *C. albicans* morphological transition, phagosomal neutralization and rupture, and pyroptosis are linked remains a topic of active research ^65, 66^.

## Membrane traffic and structural organization

Secretion plays an essential role during *C. albicans* virulence, in releasing candidalysin and a variety of proteases and lipases. In addition to using the conventional secretory pathway to secrete components into the external medium, similar to other fungi, *C. albicans* releases extracellular vesicles (EVs) ^67^, which contain cytoplasmic and moonlighting proteins, and membrane and cell wall–related proteins ^68^. A recent study elegantly showed that the EV population and composition released by *C. albicans* during growth in a biofilm are distinct from those of planktonic cells ^69^. In particular, as exogenous delivery of wild-type vesicles restores the biofilm drug-resistant phenotype and matrix composition to a subset of ESCRT (endosomal sorting complexes required for transport) mutants, it was proposed that biofilm EVs, which consist predominantly of a 30- to 200-nm diameter population, corresponding in size to exosomes, have a direct role in matrix biogenesis and carry specific cargos to confer drug resistance. The mechanism by which EVs would reach the matrix is still unclear. However, a recent work shows that AmBisome (60 to 80 nm liposomes) can traverse the cell wall ^70^, suggesting that EVs may also directly transit the cell wall.

Rapid hyphal growth requires active endocytosis to counterbalance exocytosis at the hyphal tip and recycle membrane lipids and proteins ^71, 72^. For example, recent work demonstrated that polarization of a chitin synthase to the hyphal apex in *A. nidulans* occurs by indirect endocytic recycling ^73^. Genetic analyses of loss-of-function mutants in a number of genes implicated in actin regulatory complexes, such as Pan1 ^74^ and Myo5 ^75– 77^, have also confirmed the importance of endocytosis in *C. albicans* hyphal growth, and two recent articles further point to an increased requirement for endocytosis during hyphal growth, compared with budding growth. Taking advantage of a complete collection of kinases and phosphatases, regulated via an inducible TETon promoter, Bar-Yosef *et al*. ^78^ identified a novel regulator of hyphal morphogenesis, Akl1 (related to the Ark/Prk family of kinases ^79^), whose overexpression reduced hyphal extension rates and conversely whose deletion resulted in an initial increase in hyphal extension rate. Furthermore, screening of well-characterized drug libraries allowed the identification of specific inhibitors of hyphal morphogenesis, related to piperazine ^32^. Although these drugs inhibited hyphal formation at concentrations that appear to be above safe levels, these studies raise the prospect of identifying molecules that target fungal endocytosis as potential inhibitors of *C. albicans* virulence.

Membrane/protein trafficking to the plasma membrane is mediated by vesicular transport between different cellular compartments, and small GTPases of the Arf (ADP-ribosylation factor) and Rab (Ras-related in the brain) families regulate each step of these processes ^80– 82^. The role of Arf proteins was recently investigated in hyphal growth and virulence. Of the five Arf/Arl proteins, Arf2 and Arl1 were shown to be critical for virulence in murine models for candidiasis, and Arl1 was more specifically required for oropharyngeal candidiasis ^83^. In addition, an *arf1* mutant was shown to exhibit reduced virulence in a murine systemic infection model and in macrophage killing yet this strain had a reduced growth rate and underwent cell cycle arrest ^84^. In the latter study, Arf1 was implicated as a regulator of endoplasmic reticulum (ER)–mitochondria interactions, which would directly or indirectly impact ERMES (ER–mitochondria encounter structure). Whereas Arf2 is required for viability, Arl1 is involved in hyphal extension and in restricting hyphal growth to a single site. The hyphal extension defect of the *arl1* mutant was associated with an altered distribution of the Rab GTPase Sec4 and both defects could be restored by overexpression of the Rab GTPase Ypt6 ^83, 85^, suggesting that a genetic interaction between Arl1 and Ypt6, perhaps via the GARP (Golgi-associated retrograde protein) complex ^86^, could be specifically critical for hyphal growth. In *S. cerevisiae*, analysis of trafficking mutants demonstrated that the late stage of exocytosis is particularly critical to regulate endocytosis ^87^, and more recently it was shown that Sec4 coordinated polarized exocytosis with the assembly of cortical actin patches that initiate endocytosis ^88^, indicating that this Rab GTPase is central for the balance in membrane trafficking.

Individual Rab GTPases can coordinate multiple transport pathways by recruiting effectors to different organelles ^89^, and the importance of Rab GTPases during hyphal growth has been investigated in filamentous fungi, such as *A. nidulans* and *N. crassa* ^3, 90^. However, as mentioned above, the differences in hyphal growth in these fungi, compared with *C. albicans*, raise the question as to how hyphae are organized to regulate membrane traffic in this organism ( Figure 1). Using three-dimensional electron microscopy, a high-resolution view of the *C. albicans* hyphal filament shows that the secretory pathway is organized in three distinct structural domains: sheet-like parallel membranes, shorter sheet-like membranes, and the Spitzenkörper (Spk), which is composed of a uniform population of approximately 60 vesicles that are about 70 nm in diameter ^33^. Thus, the *C. albicans* Spk appears to be simpler than that of filamentous fungi, which is composed of a heterogeneous population of vesicles ^2– 4^. Dynamic analyses of vesicle delivery to the apex suggest that short-range vesicle delivery significantly contributes to filamentous growth in *C. albicans* and that the Spk could act as a focal point for incoming secretory vesicle traffic, produced in the subapical and apex regions ^33^. These distinctions between the Spk of *C. albicans* and that of filamentous fungi might reflect differences in their function. In particular, a characteristic shape change of the Spk, from globular to crescent-like, appeared to be associated with increased extension rate in *A. nidulans*, as secretory vesicles accumulated at the Spk during phases of slow growth subsequently fused with the plasma membrane ^91^. Such a stepwise growth mode in hyphae has been shown in several filamentous fungi ^92, 93^ but thus far not in *C. albicans*.

## Conclusions

Overall, this broad range of findings in the past several years has provided both exciting novel approaches and new research directions that give us insight into the biology of this fascinating fungal pathogen. As we understand, in greater detail, the basic biology of this fungus, we now can put this new knowledge into the context of the host and the balance between commensalism and infection. Without a doubt, the advent of new technologies, in particular the combination of large-scale approaches, and effectively mixing and matching them with animal-based studies will provide powerful platforms for novel gene discovery and functional analyses in the years ahead.
